# Exosome, an important transmitter in the drug resistance of non-small cell lung cancer

**DOI:** 10.3389/fonc.2025.1539047

**Published:** 2025-05-15

**Authors:** Hongzhi Ji, Li Zhang, Lingyun Ye

**Affiliations:** ^1^ Department of Respiratory, Affiliated Hospital of Shandong Second Medical University, Weifang, Shandong, China; ^2^ Department of Gastroenterology, Affiliated Hospital of Shandong Second Medical University, Weifang, Shandong, China; ^3^ Department of Oncology, Shanghai Pulmonary Hospital & Thoracic Cancer Institute, Tongji University School of Medicine, Shanghai, China

**Keywords:** drug resistance, exosomes, chemotherapy, targeted therapy, immunotherapy

## Abstract

Recent studies have promoted new insights into the biology of non-small cell lung cancer (NSCLC) and made considerable progress in the field of treatment, including targeted therapy for driver gene mutations. Immunotherapy (IO) is another breakthrough, which has achieved amazing clinical efficacy. However, the survival status of advanced NSCLC patients is still unsatisfactory. Drug resistance is an urgent problem to be solved in almost all anti-cancer treatment schemes. Nowadays, platinum based chemotherapy remains the standard treatment for patients with driver gene negative advanced NSCLC. Previous studies have shown that the reduction of intracellular accumulation of platinum drugs, DNA damage repair and the enhancement of detoxification effect all lead to platinum resistance. The mechanisms of tyrosine kinase inhibitors (TKIs) resistance include the emergence of secondary mutation, the activation of bypass signal pathways, the abnormality of downstream signal pathways and the transformation of phenotype. The mechanisms of immune checkpoint inhibitors (ICIs) resistance are more complex. A variety of cells, cytokines and metabolites participate in it to form an immunosuppressive microenvironment, resulting in the impairment of effector T cell function. Exosomes are small molecules secreted by a variety of cells. They can carry information such as miRNA, lncRNA, and protein, and play a pivotal role in signal transduction between cells. More and more studies show that exosomes are important transmitters in lung cancer cells, which can transfer drug resistance information from drug-resistant cells to sensitive cells. However, the underling specific mechanisms need to be further explored to find a new breakthrough for overcoming drug resistance of NSCLC.

## Introduction

Non-small cell lung cancer (NSCLC) accounts for 80% of the histological types of lung cancer (1). While surgery remains the optimal treatment for early-stage patients, most are diagnosed at advanced stages and thus ineligible for resection (2). Currently, platinum-based chemotherapy serves as the standard systemic therapy for advanced NSCLC (3, 4). However, its efficacy is severely limited by intrinsic and acquired resistance—only 30% of patients respond initially, median overall survival (OS) falls below one year, and nearly all eventually develop refractory disease, culminating in poor prognosis (5, 6).

The advent of targeted therapies marked a paradigm shift. Following the 2004 discovery of epidermal growth factor receptor (EGFR) mutations, the first-generation EGFR tyrosine kinase inhibitor (TKIs) like gefitinib showed surprising efficacy in clinical trial (7, 8). Since then, a variety of driver gene mutations were gradually identified in NSCLC, such as EML4-ALK translocations and KRAS mutations (9), which has dramatically changed the treatment strategy of lung cancer. Compared to traditional chemotherapy, targeted therapy could significantly improve the survival of advanced NSCLC patients with driver gene mutations (10, 11). However, patients treated with TKIs also develop acquired drug resistance, so the duration of their clinical benefits is limited.

Immunotherapy (IO) emerged as another breakthrough in the treatment of advanced lung cancer patients, changing the landscape of NSCLC in different settings (12). Various large randomized clinical trials have shown that immune checkpoint inhibitors (ICIs) could significantly improve the survival of NSCLC patients when compared with conventional chemotherapy (13, 14), several anti-PD1/PD-L1 antibodies have been used for antitumor therapy in clinical (15). Despite remarkable clinical advances have been achieved in immunotherapy, primary and secondary resistance mechanisms restrict the durable responses of tumors to ICIs, leaving most patients with eventual disease progression.

Critically, drug resistance represents the central bottleneck across all NSCLC therapies—a multifaceted process driven by tumor-intrinsic mechanisms, microenvironmental crosstalk, and intercellular communication. Among these, exosomes have emerged as pivotal mediators. These extracellular vesicles not only facilitate tumor progression and metastasis but also orchestrate chemotherapy, targeted therapy, and immunotherapy resistance through cargo transfer (such as miRNAs, proteins) between cancer and stromal cells (16, 17).

This review systematically analyzes the resistance mechanisms underlying conventional NSCLC treatments and the deterministic role of exosomes in fostering therapeutic escape. By elucidating these pathways, we aim to guide future strategies for overcoming resistance through exosome-targeted interventions.

## Biogenesis and function of exosomes

**Figure 1:**
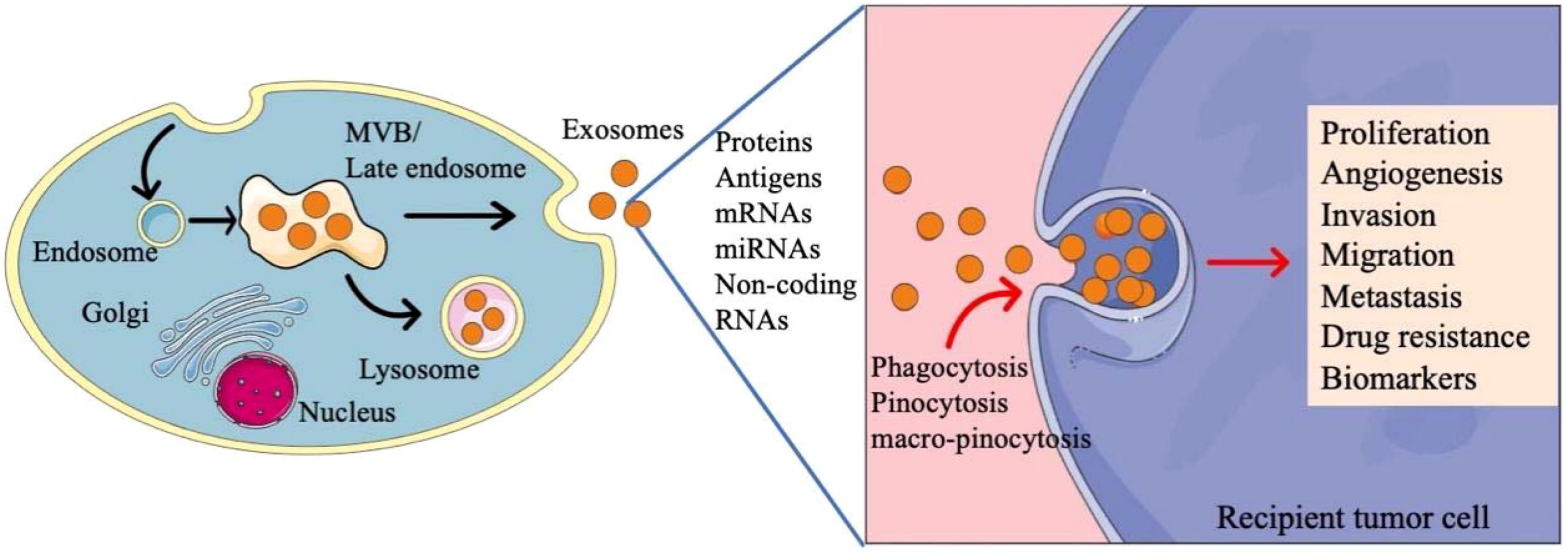
The biogenesis and function of exosomes in tumor cells. Exosomes are produced in cells through a variety of dynamic endocytosis pathways. Tumor derived exosomes contain varying quantities of molecules, participating in the regulation of cell activies.

Exosomes are 30–150 nm small molecules secreted by both normal cells and cancer cells (18), which can be isolated in many body fluids such as blood, urine, saliva, bronchoalveolar fluid, etc. (19). Exosomes are produced in cells through a variety of dynamic endocytosis pathways (**Figure 1**). Firstly, membrane proteins sprout inward through the plasma membrane and are endocytosed to form early endosomes. Then, the early endosomes mature into the late endosomes, which are known as multivesicular bodies (MVBs) (20). MVBs eventually enter the lysosome, where hydrolases and other enzymes degrade some components of MVBs and remove toxic substances. MVBs have specific surface proteins, such as CD63, lysosome related membrane proteins Lamp1 and Lamp2, so they can fuse to the plasma membrane and become exosomes to release to the extracellular environment (21, 22). Exosomes can adhere to receptor cells through integrins on the surface, then induce intracellular signals. Recipient cells take up exosomes through phagocytosis, pinocytosis, and macro-pinocytosis (23). In addition, exosomes can fuse with the plasma membrane directly to release their contents, regulating the signal transduction of the recipient cells (24).

Exosomes contain varying quantities of molecules such as microRNAs (miRNAs), messenger RNAs (mRNAs) and proteins (25). Tumor derived exosomes in tumor microenvironment have unique contents, mediating interactions between cancer cells and stromal cells, participating in tumor proliferation, angiogenesis, invasion, migration, metastasis and drug resistance (26). In addition, exosomes can also be used as biomarkers for early diagnosis and prognosis evaluation (27). Considering the regulatory role of the components of exosomes in tumor microenvironment, exosomes have been explored for the treatment of cancers. A lot of studies have reported that exosomes can be modified to deliver molecules with therapeutic effects, which will become a promising drug delivery system because of the low toxicity and inherent intercellular communication ability (28, 29).

## Role of exosomes in cisplatin resistance of NSCLC

Previous studies have shown that more than 60% of patients with NSCLC were resistant to cisplatin and carboplatin (30). In addition, these patients also showed varying degrees of resistance to other drugs, including docetaxel, gemcitabine, paclitaxel and vinorelbine (30). Therefore, almost all patients with NSCLC will finally develop resistance to chemotherapeutic drugs, even if the initial response is satisfactory. Chemoresistance is a major obstacle in the process of antitumor treatment for all patients and it is one of the main challenges in cancer management. The mechanisms related to chemoresistance are complex and have not been fully understood.

Due to the development of TKIs and ICIs and the diversification of chemotherapy schemes, the treatment options of cancer are increasing. However, cisplatin is still a highly potent anticancer drug, and its resistance mechanism is the most deeply studied in chemotherapeutic drugs at present.

Cisplatin is a kind of cell cycle nonspecific cytotoxic drug. It binds to nucleophilic groups in cells and is selectively distributed in tumor tissues. Cisplatin is hydrolyzed after entering tumor cells and then forms cisplatin-DNA complex with cell DNA. This process can destroy the normal structure of cell DNA, hinder the template effect of DNA, inhibit DNA replication and transcription (31, 32). At the same time, cisplatin affects DNA repair, induces oxidative stress, activates apoptosis, and eventually leads to tumor cell death by activating a variety of signal pathways (33).

The mechanisms of cisplatin resistance of tumor cells are multifaceted. Cisplatin can affect the expression of some transporters, thus reducing their accumulation in cells. Studies have shown that low expression of copper transporter 1 (CTR1) in lung cancer may be related to cisplatin resistance (34). Nucleotide excision repair (NER) system and mismatch repair (MMR) pathway play a key role in the repair of DNA damage caused by chemotherapy (35, 36). Abnormal expression of NER components (such as xeroderma pigmentosum group A (XPA)) and MMR related proteins (such as MutS homologue 2 (MSH2)) can enhance DNA repair and reduce the sensitivity of tumor cells to cisplatin (37, 38). Reduced glutathione (GSH), metallothionein (MT) and other nucleophilic “scavengers” in the cytoplasm can chelate cisplatin, thus reducing the accumulation and cytotoxic activity of cisplatin (39). Cisplatin can inhibit the tumor cells apoptosis and produce drug resistance by regulating PI3K/AKT, Bax/Bcl-2 and other signal pathways (40). In addition, abnormal extracellular matrix and epithelial mesenchymal transition (EMT) can promote the insensitivity of lung cancer cells to cisplatin (41, 42).

In recent years, many studies have explored the mechanisms of exosomes in cisplatin resistance, especially the miRNAs in exosomes, which play an important role in transmitting drug resistance information. The sources of these exosomes are mainly divided into two categories. Some exosomes are derived from tumor cells, while others are secreted by non-tumor cells (**Table 1**).

**Table 1:** Exosomes involved in chemoresistance of NSCLC and their potential targets.

Source	Content	Target
tumor cells derived	miR-100-5p (43, 44)	mTOR
	miR-4443 (45)	METTL3/FSP1
	miR-425-3p (46)	AKT1
	miR-197-5p (43)	–
	miR-642a-3p (43)	–
	miR-27b-3p (43)	–
	PKM2 (47)	Bcl2
Non- tumor cells derived		
CAFs	miR-103a-3p (48)	BAK1
CAFs	miR-130a (49)	–
BMSCs	miR-193a (50)	LRRC1

mTOR, mammalian target of rapamycin; PKM2, pyruvate kinase M2; CAFs, cancer-associated fibroblasts; BMSCs, bone marrow-derived mesenchymal stem cells.

It was found that when lung cancer cells A549 were exposed to cisplatin, they could release more exosomes, and when these exosomes were co-cultured with other A549 cells, their resistance to cisplatin increased. It indicated that A549 cells could release exosomes for intercellular signal communication and realize the transmission of intercellular drug resistance (51). Five microRNAs with the most significant expression difference were found in the exosomes of A549 cells and cisplatin resistance A549 cells (A549/DDP), they were miR-27b-3p, miR-100-5p, miR-197-5p, miR-4443 and miR-642a-3p. Target gene prediction and pathway analysis suggested that these microRNA might be important regulators in the drug resistance of cisplatin (43). There was a study found that compared with A549 sensitive strain, the expression of miR-100-5p in the exosomes of A549/DDP cells was significantly downregulated, while the expression of mammalian target of rapamycin (mTOR) in recipient cells was regulated by miR-100-5p. Therefore, exosomes from cisplatin resistant lung cancer cells could change the sensitivity of other cells by regulating mTOR through miR-100-5p (44). While another study showed that the level of miR-4443 in cisplatin resistant NSCLC tumor tissue-derived exosomes was upregulated. And mechanistic studies indicated that exosomal miR-4443 could participate in the regulation of cisplatin resistance of NSCLC cells, and METTL3/FSP1-mediated ferroptosis might be the potential mechanism (45). After cisplatin stimulation, the expression of miR-425-3p in exosomes was induced by c-Myc-mediated transactivation. Further studies showed that exosomal miR-425-3p promoted the activation of autophagy by targeting AKT1, which eventually reduced the sensitivity of recipient cells to cisplatin (46). A study found that hypoxia induced NSCLC cell-derived exosomal pyruvate kinase M2 (PKM2, a rate-limiting enzyme in glycolysis) could promote the glycolysis of NSCLC cells, reduce cisplatin induced reactive oxygen species (ROS), and inhibit apoptosis through PKM2-Bcl2 pathway. In addition, exosomal PKM2 induced by hypoxia could reprogram cancer-associated fibroblasts (CAFs) to create an acidic environment, thus promoting cisplatin resistance of NSCLC cells (47).

Non-tumor cells derived exosomes are also important transmitters of intercellular drug resistance information. It was found that MiR-103a-3p was highly expressed in CAFs and CAFs exosomes in NSCLC. When CAFs exosomes were added to culture medium of NSCLC cells, the expression of miR-103a-3p in exosomes of NSCLC cells increased. *In vitro* experiments showed that exosomal miR-103a-3p derived from CAFs could inhibit cisplatin induced apoptosis and promote resistance to cisplatin in NSCLC cells, which has also been confirmed by mouse tumorigenesis assay *in vivo*. While the RNA-binding protein pumilio homolog 2 (PUM2) could facilitate miR-103a-3p packaging into CAFs derived exosomes in cytoplasm and nucleus. Further experiments showed that miR-103a-3p promoted the resistance of NSCLC cells to cisplatin by downregulating BAK1 (48). In addition, another study showed that miR-130a in CAFs derived exosomes could also promote the resistance of NSCLC cells to cisplatin, and PUM2 was also involved in the packaging process of miR-130a into exosomes (49). The expression of miR-193a in bone marrow-derived mesenchymal stem cells (BMSCs) derived exosomes was increased, which could inhibit the proliferation and migration of NSCLC cisplatin resistant cells. The BMSCs derived exosomal miR-193a could also promote apoptosis and reduce the cisplatin resistance of NSCLC cells. And the downregulation of LRRC1 expression caused by miR-193a played a regulatory role in the mechanism of cisplatin resistance (50).

## Role of exosomes in TKIs resistance of NSCLC

Two classic oncogene mutations in NSCLC are epidermal growth factor receptor (EGFR) mutation or anaplastic lymphoma kinase (ALK) chromosome rearrangement (the most common one is EML4-ALK fusion) (52). These two gene mutations have become the standard and routine detection items of advanced lung adenocarcinoma. In various randomized phase III clinical trials, EGFR and ALK TKIs have always shown higher efficacy than chemotherapy, making targeted therapy the standard treatment for advanced NSCLC with such gene mutations (53, 54). However, drug resistance remains a pervasive challenge in clinic, patients will eventually get progress during targeted therapy. Therefore, in this review, we mainly focus on the molecular mechanisms of drug resistance of EGFR and ALK TKIs.

According to the response of the tumor to the initial treatment, drug resistance can be divided into two types. One is primary drug resistance, who have no response at all to the treatment. The other is acquired drug resistance. Such patients may have a complete or partial response at first, but eventually have no response over time (55). The primary resistance of NSCLC to EGFR TKIs is mainly related to wild-type EGFR. The activation mutation of KRAS or BRAF and the loss of function of apoptotic protein BIM will lead to primary drug resistance (56–58). Some rare EGFR mutations can also reduce the sensitivity of tumors to TKIs, such as small insertions or duplications in exon 20 (59). Fibroblast growth factor (FGF), hepatocyte growth factor (HGF) and neuregulin1 (NRG1) in tumor microenvironment are involved in the regulation of primary drug resistance by regulating Ras/MAPK or PI3K/Akt signaling pathway (60). In addition, the activation of NF-κB signaling is also identified as one of the mechanisms of primary resistance to EGFR TKIs (61).

**Figure 2:**
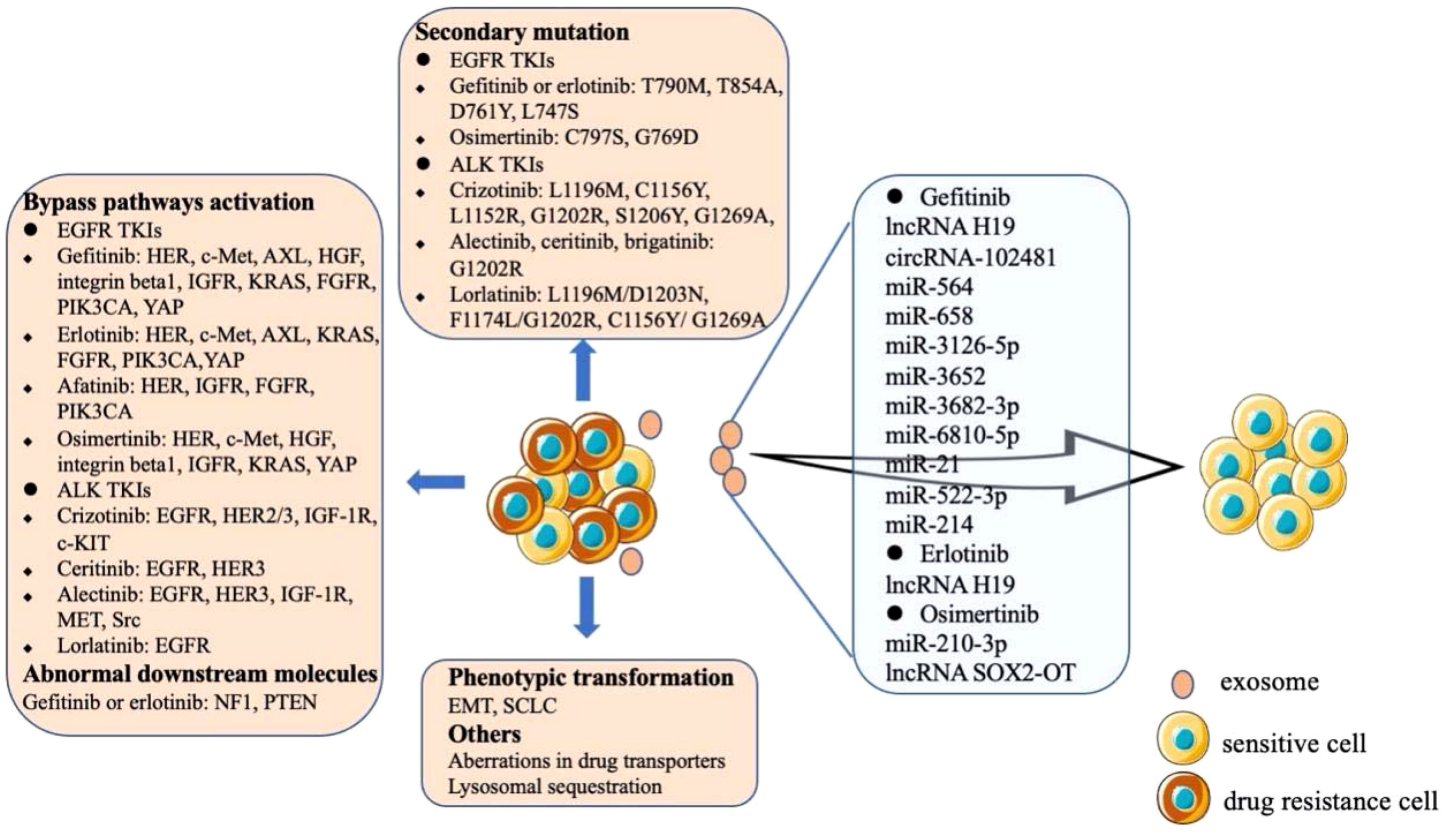
Mechanisms of acquired drug resistance of EGFR and ALK TKIs. The resistance mechanisms of TKIs mainly include the emergence of secondary mutations, bypass activation and phenotypic transformation. The exosomes secreted by drug-resistant cells contain special cytokines, which are transmitted to sensitive cells to make them resistant to TKIs.

In recent years, extensive efforts have been made to clarifying the mechanisms of acquired drug resistance of TKIs (**Figure 2**). The acquired resistance of the first-generation EGFR TKIs (gefitinib or erlotinib) is mainly mediated by the development of T790M mutation, which occurs in 50-65% of EFGR mutation and TKIs resistant patients (62), other secondary mutations including T854A, D761Y and L747S (63). The first mutation of resistance to second-generation EGFR-TKI (afatinib) is also T790M mutation, and the secondary mutation of resistance to third generation EGFR-TKI (osimertinib) is EGFR C797S or G796D mutation (64). For the first generation ALK-TKI crizotinib resistant patients, ALK kinase domain mutations are the most common mechanisms, including L1196 M, C1156Y, L1152 R, G1202R, S1206Y and G1269A mutations (65, 66). And G1202R is the most common mutation in patients resistant to second-generation ALK-TKIs (alectinib, ceritinib, brigatinib) (67). Unlike the first or second generation ALK-TKIs, most lorlatinib resistant patients have multiple ALK mutations, such as L1196M/D1203N, F1174L/G1202R and C1156Y/G1269A (68).

Another mechanism of TKIs resistance is the aberrated activation of the bypass pathways (**Figure 2**). The aberrance of other members of HER family, amplification of c-Met, overexpression of AXL, overexpression of HGF, overexpression and activation of integrin beta1, and the abnormality of IGFR can affect the PI3K/AKT, MAPK, ERK, or NF-κB signaling pathways to induce EGFR TKIs resistance (63). Abnormal expression of downstream molecules may also lead to resistance to gefitinib or erlotinib, such as the aberrant expression of NF1 and the loss of PTEN (63). Bypass signaling tracks associated with ALK-TLIs resistance including the abnormal of EGFR, HER2/3, IGF-1R, c-KIT, etc. (**Figure 2**) (69). The other two rare mechanisms of acquired TKIs resistance are the phenotypic change of NSCLC via EMT and the histological transformation of NSCLC into SCLC (63, 69). Some drug transporters (ABCB1/PGP and ABCG2/BCRP) and lysosomal sequestration may also be involved in erlotinib and gefitinib resistance (64). In addition, tumor heterogeneity is a common phenomenon. Generally, TKIs sensitive cells and acquired drug-resistant tumor cells are mixed. When the sensitive cells are killed, the drug-resistant cells proliferate rapidly and become the dominant cell group, further enhancing the drug resistance (69).

The studies of exosomes in drug resistance of TKIs mainly focus on gefitinib, erlotinib and osimertinib. It was found that the level of lncRNA H19 in gefitinib resistant cells was higher than that in sensitive cells. In addition, knockout of lncRNA H19 increased the sensitivity of cells to gefitinib. Further studies confirmed that lncRNA H19 could be encapsulated in exosomes and transferred to sensitive cells to induce gefitinib resistance, which was specifically mediated by heterogeneous nuclear ribonucleoprotein A2B1 (hnRNPA2B1), an RNA binding protein that controls the loading of RNA into exosomes (70). Tumor derived exosomal circRNA-102481 could enhance the expression of ROR1 and promote gefitinib resistance (71). A microRNA analysis of exosomes showed that miR-564, miR-658, miR-3126-5p, miR-3652, miR-3682-3p and miR-6810-5p were significantly up-regulated in the exosomes of gefitinib resistant cells. Further studies proved that exosomal miR-564 and miR-658 from gefitinib resistant NSCLC cells could induce drug resistance in sensitive cells (72). Studies have shown that tumor-derived exosomal miR-21 and miR-522-3p could participate in gefitinib resistance by regulating PI3K/Akt signaling pathway, while exosomal miR-214 could confer gefitinib resistance via Bax/Bcl2 signaling (73–75). Exosomal lncRNA H19 could also regulate the expression of autophagy-related protein 7(ATG7) by targeting miR-615-3p, thus affecting the drug resistance of NSCLC cells to erlotinib (76). A study demonstrated that exosomes containing wild-type EGFR protein were internalized by EGFR mutant cancer cells through clathrin-dependent endocytosis, and then the downstream PI3K/Akt and MAPK signaling pathways were activated by the wild-type EGFR protein, thus triggering osimertinib resistance (77). It was found that miR-210-3p was highly expressed in the exosomes of osimertinib resistant cells. What’s more, exosomal miR-210-3p could directly promote EMT of tumor cells and resistance to osimertinib (78). In addition, NSCLC cell line H1975 could transfer lncRNA SOX2-OT into macrophages through exosomes. LncRNA SOX2-OT facilitated the expression of Smads by sponging miR-627-3p and induced macrophages M2 polarization to aggravate the drug resistance of cancer cells to osimertinib (79).

To clarify the role of exosomes in NSCLC drug resistance, a comparison with classical mechanisms, such as gene mutations and drug efflux pumps, is instructive. Mutations like EGFR T790M primarily confer resistance by decreasing the binding affinity for tyrosine kinase inhibitors, thus limiting therapeutic efficacy (73). Meanwhile, drug efflux transporters such as ABCB1 and ABCG2 reduce drug accumulation inside tumor cells through active drug export, resulting in treatment failure (80). These traditional resistance mechanisms are typically cell-intrinsic, affecting individual cancer cells, and generally irreversible once established. By comparison, exosomes provide a distinctive mechanism involving intercellular communication. They can transfer resistance-associated molecules—including miRNAs and proteins—from resistant cells to sensitive cells, thus propagating resistance throughout diverse tumor populations (73). Additionally, exosomes modulate the tumor microenvironment and mediate immune evasion, which are distinct processes not typically associated with traditional mutation or efflux-based resistance mechanisms (81). Clinically, the presence of exosomal contents in biofluids allows for non-invasive monitoring of treatment response. Furthermore, exosome secretion, uptake, and cargo packaging are processes amenable to therapeutic targeting. Therefore, exosomes hold promise not only as biomarkers for resistance but also as novel therapeutic targets, underscoring their increasingly recognized and multifaceted role in NSCLC drug resistance.

## Role of exosomes in ICIs resistance of NSCLC

**Figure 3:**
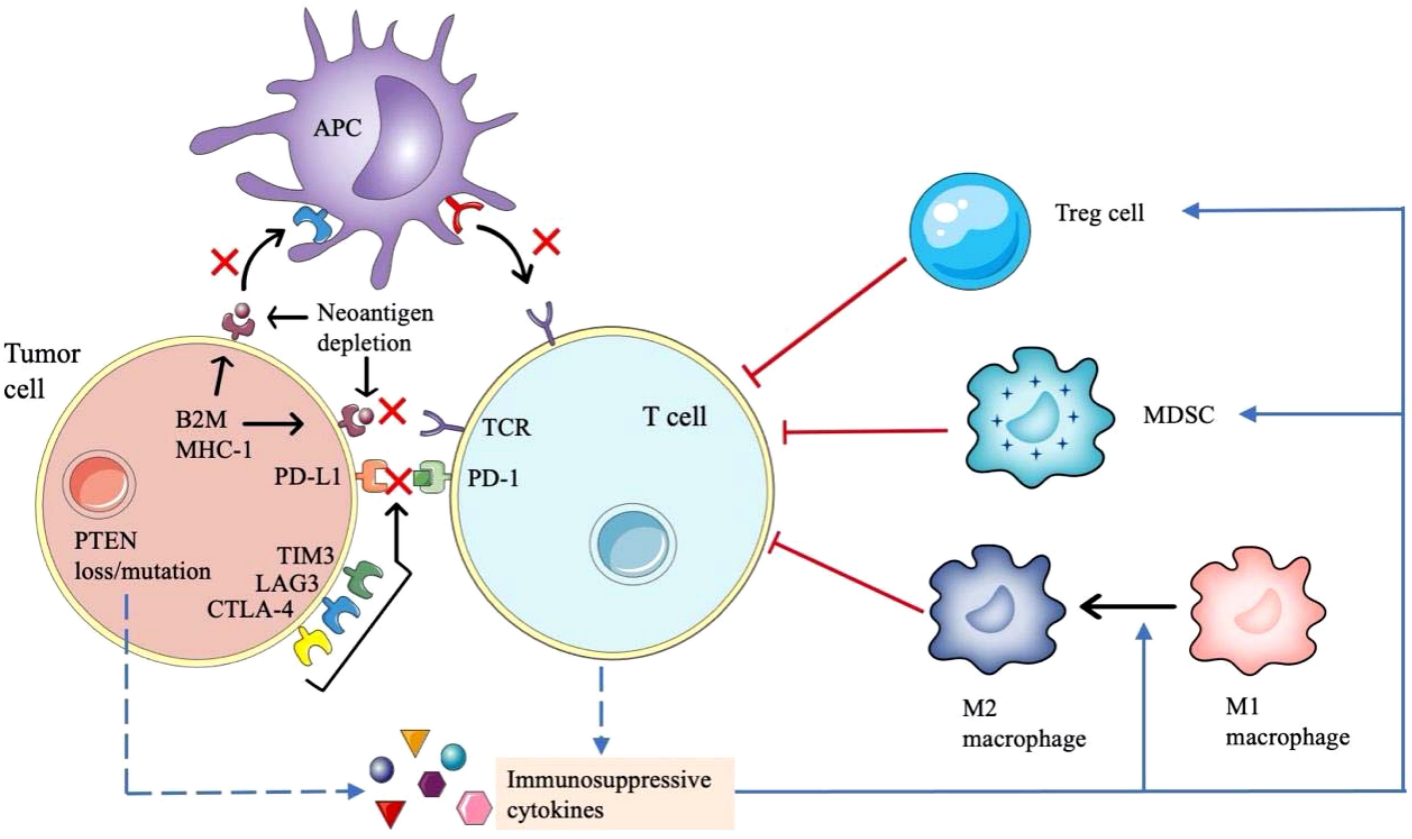
Mechanisms of drug resistance of immune checkpoint inhibitors in NSCLC. The mechanism of immunotherapy resistance is complex. Loss of antigen expression or presentation defect of tumor cells, abnormal signaling pathways in tumor cells and upregulation of non-PD-1 / PD-L1 immune checkpoints will affect the interaction between tumor cells, antigen presenting cells and T cells. In addition, many immunosuppressive cytokines will inhibit the antitumor activity of T cells.

Drug resistance of ICIs can also be divided into primary resistance and acquired resistance. Statistics show that less than 30% of NSCLC patients respond to ICIs, and most patients suffer primary resistance (82). The emergence of ICIs resistance is a complex, dynamic and interdependent process, which is closely related to many tumor and host factors (**Figure 3**).

The efficacy of ICIs depends on the formation of tumor new antigens. If the tumor specific antigens are less expressed and their immunogenicity become weak, it will be not enough to activate primitive T cells. And if a neoantigen is structurally like an immune tolerance antigen or an autoantigen, it will be difficult to be recognized by antigen-presenting cells (APCs) and activate T cells, which will lead to the drug resistance to PD-1/PD-L1 inhibitors (83). Class I MHC, β-2 microglobulin (β2M), large multifunctional proteinase (LMP) and transporter associated with antigen processing (TAP) are important components of tumor antigen processing and presentation devices, resistance to PD-1/PD-L1 inhibitors also occurs when the genes encoding them become abnormal. For example, the loss of β2M expression leads to the impaired expression of class I MHC molecules on the surface of APCs, resulting in impaired antigen presentation and finally immune tolerance (84). Furthermore, the downregulation of HLA class I molecules may also be related to ICIs resistance (85).

Several factors lead to the inadequate function of tumor specific T cells and reduce the clinical effect of PD-1/PD-L1 inhibitors. The expression of many co-inhibitory receptors, such as cytotoxic T lymphocyte antigen 4 (CTLA-4), T cell immunoglobulin and mucin domain-3 (TIM3) and lymphocyte activation gene 3 (LAG3) participate in immune escape and affect the antitumor effect of T cells (86). Emerging evidence suggest that T cell exhaustion is associated with epigenetic changes manifested as immune dysfunction and continuous expression of surface inhibitory receptors, making it difficult to function as normal effector T cells (87).

A variety of cells and cytokines in tumor microenvironment (TME) form an immunosuppressive state, which can diminish the therapeutic efficacy of PD-1/PD-L1 inhibitors. Regulatory T cells (Tregs) can inhibit the function of effector T cells by secreting some inhibitory cytokines (88, 89). The removal of Tregs in the TME can enhance the antitumor effect of PD-1/PD-L1 inhibitors (90). MDSCs are the main regulatory factors of immune response under various pathological conditions, which can promote angiogenesis, tumor invasion and metastasis. The presence of MDSCs in TME will reduce the effect of IO (91). Tumor associated macrophages (TAMs), including M1 macrophages and M2 macrophages, another class of cells that regulate the immune environment against tumors (92). Immunosuppressive cytokines such as CCL5, CCL7 and CXCL8, can aggregate MDSCs and Tregs into the TME (93). In addition, indoleamine 2,3-dioxygenase (IDO) produced by tumor or immune cells can improve the production and activity of Tregs and MDSCs, produce immunosuppressive metabolites to influence the function of effector T cells (94). Some gene mutations are associated with immunosuppression. PTEN gene deletion leads to the upregulation of CCL2, hypoxia inhibitory factor 1 (HIF-1) and vascular endothelial growth factor (VEGF), which leads to the transformation of macrophages from M1 type to M2 type, resulting in negative immune regulation (95). KRAS mutation will cause the loss of STK11/LKB1, and then T cell suppressor neutrophils are recruited, resulting in the reduction of T cells infiltration (96, 97).

Tumor cells can produce exosomes rich in cancer promoting components, such as immunosuppressive proteins like PD-L1, regulating immune response and promoting drug resistance (98). PD-L1 derived from tumor exosomes presented both on the surface and within exosomes, and exosomes can transport PD-L1 to other cells with low or no expression of PD-L1 and may bind to PD-1 (99). Insoluble PD-L1 expressed on plasma/serum exosomes is associated with disease progression of NSCLC (100). Previous study found that exosomes containing PD-L1 isolated from NSCLC patients could reduce the production of IFN-γ and interleukin-2 (IL-2), inhibiting the activity of CD8+ T cells, this effect was more significant in exosomes with high level of PD-L1 (101). What’s more, exosomes with high levels of PD-L1 could also induce apoptosis of CD8+ T cells through PD-L1/PD-1 interaction (102). So far, there are few studies on exosomes in ICIs resistance of NSCLC. It is necessary to further explore and clarify their role in immune regulation, to provide new theoretical basis for ICIs resistance, and look for potential therapeutic breakthroughs.

Building upon the mechanistic distinctions outlined above, we further explore the unique clinical implications of exosome-mediated resistance in NSCLC. Exosome-mediated resistance differs from traditional drug resistance mechanisms in NSCLC, such as gene mutations, drug efflux pumps, EMT, and tumor heterogeneity. Generally, mechanisms like gene mutations or drug efflux involve intrinsic cellular changes, often irreversible and restricted to single tumor cells. In contrast, exosomes can deliver resistance-associated molecules such as miRNAs and proteins from resistant cells to sensitive ones, enabling the horizontal spread of resistance throughout tumor populations (103).

Moreover, exosomes have additional roles in modifying the tumor microenvironment and facilitating immune escape, features rarely seen with mutations or efflux pump-related resistance (104). Clinically, exosomes can be detected in patients’ body fluids, providing a non-invasive way to monitor drug resistance and disease progression dynamically. Importantly, various steps involved in exosome biology—including secretion, uptake, and cargo packaging—can be therapeutically targeted, offering novel strategies for overcoming drug resistance beyond conventional approaches (105).

## Emerging exosome-based combination strategies in NSCLC

Recent studies suggest that exosome-targeting approaches may complement existing NSCLC therapies by enhancing drug sensitivity, overcoming resistance, or serving as biomarkers. **Table 2** provides representative examples of such strategies under preclinical or exploratory investigation (105–108).

**Table 2:** Potential combination therapies involving exosome-targeting and standard NSCLC treatments.

Strategy	Description	Reference
Exosomal paclitaxel + Cisplatin	Oral exosomal paclitaxel improves efficacy and reduces toxicity when combined with cisplatin.	(106)
Exosome secretion inhibitor (GW4869) + Chemotherapy	Enhances response to cisplatin by blocking exosome-mediated resistance	(107)
Gefitinib + Exosome modulation	Inhibiting tumor macrophage-derived exosomes improves TKI sensitivity.	(108)
ICIs + Exosomal miRNA-based biomarkers	Exosomal miRNAs used to monitor ICI response and resistance development.	(105)

## Conclusion

Drug resistance is an inevitable thorny problem in the clinical treatment of NSCLC. Whether it is the classical chemotherapy, targeted therapy or immunotherapy, the emergence of drug resistance limits the clinical efficacy, and its molecular mechanisms are multifactorial and complex. There is increasing evidence that exosomes are involved in the drug resistance process of NSCLC, which can transmit information between cells though the miRNAs or proteins in it. However, further exploration of tumor microenvironment after lung cancer treatment is still needed to overcome the issue of drug resistance.
